# Self-medication with antibiotics in rural population in Greece: a cross-sectional multicenter study

**DOI:** 10.1186/1471-2296-11-58

**Published:** 2010-08-08

**Authors:** Eystathios Skliros, Panagiotis Merkouris, Athanasia Papazafiropoulou, Aristofanis Gikas, George Matzouranis, Christos Papafragos, Ioannis Tsakanikas, Irene Zarbala, Alexios Vasibosis, Petroula Stamataki, Alexios Sotiropoulos

**Affiliations:** 1 3rd Department of Internal Medicine and Center of Diabetes, General Hospital of Nikaia "Ag. Panteleimon" - Piraeus, Greece, 3 D.Mantouvalou Street, GR-184 54 Nikaia, Greece

## Abstract

**Background:**

Self-medication is an important driver of antimicrobial overuse as well as a worldwide problem. The aim of the present study was to estimate the use of antibiotics, without medical prescription, in a sample of rural population presenting in primary care in southern Greece.

**Methods:**

The study included data from 1,139 randomly selected adults (545 men/594 women, mean age ± SD: 56.2 ± 19.8 years), who visited the 6 rural Health Centres of southern Greece, between November 2009 and January 2010. The eligible participants were sought out on a one-to-one basis and asked to answer an anonymous questionnaire.

**Results:**

Use of antibiotics within the past 12 months was reported by 888 participants (77.9%). 508 individuals (44.6%) reported that they had received antibiotics without medical prescription at least one time. The major source of self-medication was the pharmacy without prescription (76.2%). The antibiotics most frequently used for self-medication were amoxicillin (18.3%), amoxicillin/clavulanic acid (15.4%), cefaclor (9.7%), cefuroxim (7.9%), cefprozil (4.7%) and ciprofloxacin (2.3%). Fever (41.2%), common cold (32.0%) and sore throat (20.6%) were the most frequent indications for the use of self-medicated antibiotics.

**Conclusion:**

In Greece, despite the open and rapid access to primary care services, it appears that a high proportion of rural adult population use antibiotics without medical prescription preferably for fever and common cold.

## Background

Since the introduction of penicillin, 60 years ago, antibiotics have played an important and crucial role in the treatment of infectious diseases, especially those caused by bacteria. However, the inappropriate use of those drugs has led to the phenomenon of antimicrobial resistance, which is becoming a worldwide public health problem [1,2]. Recent studies in Europe, including Greece, have showed high rates of outpatient antibiotic use and resistance [2-5]. In particular, a shift from the old narrow-spectrum antibiotics to the new broad-spectrum antibiotics was observed [3]. They also recorded seasonal fluctuations with heightened winter peaks in countries with high yearly use of antibiotics [3]. It is noteworthy, that our country had one of the highest uses of antimicrobial agents in ambulatory care [5].

Self-medication is an important driver of antimicrobial overuse, especially, in low- and middle-income countries, where antibiotics are easily obtained over the counter [1,6,7]. Studies showed that the prevalence of actual self-medication was high in eastern and southern Europe and low in northern and western Europe [8-10]. This studies showed that the most common reasons for self-medication were throat symptoms and bronchitis [8,10] while the main medication sources were pharmacies and medication leftover from previous prescriptions [8-10]. In Greece, the limited data concerning urban population indicate that the prevalence of self-medication is high [11,12]. However, data about self-medication in rural areas are lacking. Therefore, the aim of the present study was to evaluate the prevalence of self-medication with antibiotics in rural population in Greece.

## Methods

### Population

The study included data from 1,139 adults (545 men/594 women, mean age ± SD: 56.2 ± 19.8 years), who visited the 6 rural Health Centres of southern Greece, between November 2009 and January 2010. Physicians of the above Health Centres carried out the study. Physicians asked each consecutive patient to fill an anonymous questionnaire and to return it at the practice. The refusal rates were low. The questionnaire included items relating to demographic characteristics, overall use of antibiotics and self-medication with antibiotics. In particular, information about the type of antibiotics, the sources of self-medication, the symptoms for which the drugs were reportedly used, and duration of use were collected.

The study was conducted in accordance with the 2004 amendment of the Declaration of Helsinki, the guidelines for Good Epidemiological Practice [13], and local regulatory requirements. The protocol was approved by the local ethics committee in each study area.

### Statistical Analysis

Statistical analysis was preformed using programs available in the SPSS statistical package (SPSS 15.0, Chicago, USA). All variables were tested for normal distribution of the data. Data are shown as mean ± SD. A chi-square test was used for categorical variables. P < 0.05 (two-tailed) was considered statistically significant.

## Results

Use of antibiotics within the past 12 months was reported by 888 participants (77.9%). 508 individuals (44.6%) reported that had received antibiotics without medical prescription at least one time in the past 12 months. The major source of self-medication was the pharmacy without prescription (76.2%) followed by leftover medications at home (15.3%) and drugs obtained from relatives or friends (7.2%).

The most frequently self-medicated antibiotics were amoxicillin (18.3%), amoxicillin/clavulanic acid (15.4%), cefaclor (9.7%), cefuroxim (7.9%), cefprozil (4.7%) and ciprofloxacin (2.3%). Fever (41.2%), common cold (32.0%) and sore throat (20.6%) were the most frequent indications for their use.

Only 9.1% (57/630) of the participants who did not report self-medication with antibiotics, had stored drugs at home compared to 49.2% (250/508) of the participants who reported self medication (P < 0.001). Finally, 31.5% of the participants reported earlier discontinuation of antibiotics when symptoms improved.

## Discussion

Our results showed that the prevalence of self-medication with antibiotics in rural population in southern Greece was high. Our results are comparable to those of Jordan were 46% of patients reported antimicrobial self-medication [6]. However, antimicrobial drug self-medication prevalence varies widely among different European regions. Studies in Denmark and Spain showed that self drug consumption was 3% and 11% respectively [14,15]. In Malta and Lithuania the prevalence of self-medication was 19% and 22% respectively [7,16]. A prospective survey of emergency department patients in the USA established that 17% of patients had taken leftover antibiotics without consulting a physician, most commonly for a cough (11%) or sore throat (42%) [17]. A recent study in Europe reported that Greece had one of the highest outpatient antibiotic uses in Europe with cephalosporins and macrolides being the most frequently used antibiotics [3].

Substantial variation in the prevalence rates of antimicrobial drug self-medication among the European regions suggests that socioeconomic factors play a role, as do disparities in health care systems such as reimbursement policies, access to health care, and drug dispensing policies [18]. Another factor is the acquisition of antimicrobial drugs from pharmacies without prescription, which occurred most frequently in eastern and southern European countries [18].

Although most of the responsibility regarding inappropriate antibiotic use belongs to the physicians' prescribing practices, several studies found that patients, too, contribute to inappropriate antibiotic usage [15,18]. Self-medication with antibiotics is possible via several sources: a) they are legally available over the counter, b) antibiotics initially prescribed by physicians are saved and subsequently used without medical consultation, c) antibiotics are obtained through friends or relatives, and d) they can be acquired via Internet [15,18].

In the present study the major source of self-medication was the pharmacy. It must be mentioned that in Greece law still allows patients to obtain antibiotics from the pharmacists without any medical prescription. A study by Contopoulos-Ioannidis et al., reported that 77% of Greek pharmacists offered antibiotics without a medical prescription [19]. The same study showed that most of pharmacists offered expensive broad-spectrum antibiotics. Antibiotics were most frequently offered for treatment of patients with symptoms that were suggestive of a common cold [19].

## Conclusions

In Greece, despite the open and rapid access to primary care services, it appears that a high proportion of rural adult population prefers to use antibiotics without medical prescription. The high prevalence of self-medication with antibiotics in rural population in Greece emphasizes the role of the primary care physician who should advise patients about the correct use of the prescribed antibiotics. Another important intervention to reduce the major problem of self-medication with antibiotics in Greece should be legislative changes banning unregulated sale of antibiotics without medical prescription. Finally, efforts like the European Antibiotic Awareness Day [20] emphasize the importance of using antibiotics responsibly by reducing their unnecessary use and encourage people to follow their doctor's instructions on how to take antibiotics in the appropriate way, especially in children.

## Competing interests

The authors declare that they have no competing interests.

## Authors' contributions

AG, GM, CP, IT, IZ, AV and PS participated in the collection of the data. ES, PM, AP and AS participated in the design of the study and performed the statistical analysis and drafted the manuscript. All authors read and approved the final manuscript.

## Pre-publication history

The pre-publication history for this paper can be accessed here:

http://www.biomedcentral.com/1471-2296/11/58/prepub
